# Actionable lessons for the US COVID vaccine program

**DOI:** 10.1186/s13584-021-00452-2

**Published:** 2021-02-19

**Authors:** Gary L. Freed

**Affiliations:** Department of Pediatrics, University of Michigan, Ann Arbor, MI USA

**Keywords:** COVID-19, Vaccination, Immunization, Policy

## Abstract

When attempting to provide lessons for other countries from the successful Israeli COVID-19 vaccine experience, it is important to distinguish between the modifiable and non-modifiable components identified in the article by Rosen, et al. Two specific modifiable components included in the Israeli program from which the US can learn are (a) a national (not individual state-based) strategy for vaccine distribution and administration and (b) a functioning public health infrastructure. As a federal government, the US maintains an often complex web of state and national authorities and responsibilities. The federal government assumed responsibility for the ordering, payment and procurement of COVID vaccine from manufacturers. In designing the subsequent steps in their COVID-19 vaccine distribution and administration plan, the Trump administration decided to rely on the states themselves to determine how best to implement guidance provided by the Centers for Disease Control and Prevention (CDC). This strategy resulted in 50 different plans and 50 different systems for the dissemination of vaccine doses, all at the level of each individual state. State health departments were neither financed, experienced nor uniformly possessed the expertise to develop and implement such plans. A national strategy for the distribution, and the workforce for the provision, of vaccine beyond the state level, similar to that which occurred in Israel, would have provided for greater efficiency and coordination across the country. The US public health infrastructure was ill-prepared and ill-staffed to take on the responsibility to deliver > 450 million doses of vaccine in an expeditious fashion, even if supply of vaccine was available. The failure to adequately invest in public health has been ubiquitous across the nation at all levels of government. Since the 2008 recession, state and local health departments have lost > 38,000 jobs and spending for state public health departments has dropped by 16% per capita and spending for local health departments has fallen by 18%. Hopefully, COVID-19 will be a wakeup call to the US with regard to the need for both a national strategy to address public health emergencies and the well-maintained infrastructure to make it happen.

The article by Rosen et al. [1] outlines several important components of the Israeli health system that contributed to the success of the initial phases of the COVID-19 vaccination program. When attempting to provide lessons for other countries from this experience, it is important to distinguish between the modifiable and non-modifiable components identified in the article. Although it is important to note the non-modifiable components that played a role in the achievement of the Israeli program, such as small geographic size and relatively small population, they do not provide the opportunity for learning that can lead to effective change and improvement in other nations.

Fortunately, Rosen and colleagues also provide several modifiable components of the Israeli experience that can serve as a framework for countries currently struggling with their COVID-19 immunization programs. With specific regard to the United States, I believe there are two components that stand out as important lessons to be learned and for which both short and long term action can be taken. These actions could directly impact the effectiveness of the current COVID-19 vaccine program and increase the readiness and capacity of the US to address any future pandemic. The two components are (a) a national (not individual state-based) strategy for vaccine distribution and administration and (b) a functioning public health infrastructure, potentially operationalized through a combination of components of the public, private and not-for-profit sectors in a coordinated mechanism as needed.

As a federal government, the US maintains an often complex web of state and national authorities and responsibilities. The fundamental authority of the states in many matters stems from the 10th Amendment to the US Constitution: “The powers not delegated to the United States by the Constitution, nor prohibited by it to the States, are reserved to the States respectively, or to the people” [2]. Originally, this amendment was designed to prevent the growth of an overly powerful central government and is the origin of political efforts to maintain the authority of states in a variety of domains [3]. Over time, this clause has resulted in significant state authority for many matters, including several aspects of health policy. The degree of state autonomy vs. national control is a common political flashpoint in the US [4].

The federal government assumed responsibility for the ordering, payment and procurement of COVID vaccine from manufacturers. However, the formula for the amount of vaccine allocated to specific states remains opaque [5]. Further, in designing the subsequent steps in their COVID-19 vaccine distribution and administration plan, the Trump administration decided to rely on the states themselves to determine how best to implement guidance provided by the Centers for Disease Control and Prevention (CDC) [6]. This strategy fulfilled two purposes. First, it sought to remove from the federal government any responsibility for the actual organization of administration of vaccines beyond the state level and therefore any potential blame for problems that might ensue. Second, it allowed the Trump administration both to champion state authority, a pillar of his COVID strategy to date, and to criticize progress in specific states with governors he deemed as political opponents.

This strategy resulted in 50 different plans and 50 different systems for the dissemination of vaccine doses, all at the level of each individual state. State health departments were neither financed, experienced nor uniformly possessed the expertise to develop and implement such plans [7]. As a result, there was tremendous variability in the process resulting in misinformation, confusion and a lack of confidence in the vaccine program among the public. A national strategy for the distribution, and the workforce for the provision, of vaccine beyond the state level, similar to that which occurred in Israel, would have provided for greater efficiency and coordination across the country.

The second modifiable component is the development and maintenance of a strong, well-trained public health infrastructure. The US public health infrastructure was ill-prepared and ill-staffed to take on the responsibility to deliver > 450 million doses of vaccine in an expeditious fashion, even if supply of vaccine was available. In the US, public health operates at a national, state and local level. The degree of coordination among those levels is highly variable across the nation. However, the failure to adequately invest in public health has been less variable, and is ubiquitous across the nation. Although investment in public health has not been a priority for decades, the problem has only grown more acute in recent years. Since the 2008 recession, state and local health departments have lost > 38,000 jobs, resulting in a skeletal workforce with insufficient staffing to meet many everyday needs, much less that of the increased demands of a population-wide immunization program [8].

The public health infrastructure has also suffered from decreasing in funding for more than a decade, having an impact on the degree of preparedness it can maintain. Just since 2010, spending for state public health departments has dropped by 16% per capita and spending for local health departments has fallen by 18% [9]. This is in addition to the cuts made directly in response to the recession of 2008. Dr. Robert Redfield, the immediate past director of the CDC, said in an interview in April 2020 that his “biggest regret” was “that our nation failed over decades to effectively invest in public health” [8]. More than three-quarters of Americans live in states that spend less than $100 per person annually on public health with half of those funds on average going to local health departments.

In contrast to the US, the article by Rosen and colleagues points to a highly developed and motivated public health infrastructure guiding the success of the Israeli immunization program. Of significance is the coordinated role of both public agencies and a not-for-profit health plans in delivering public health services in Israel.

## Conclusions

Governments often can underfund preventive and public health efforts without consequence until a serious event occurs that lays bare the implications of that neglect. Hopefully, COVID-19 will be a wakeup call to the US with regard to the need for both a national strategy to address public health emergencies and the well-maintained infrastructure to make it happen.

## Data Availability

N/A
